# Fibrinogen protects neutrophils from the cytotoxic effects of histones and delays neutrophil extracellular trap formation induced by ionomycin

**DOI:** 10.1038/s41598-020-68584-0

**Published:** 2020-07-16

**Authors:** Matthew Locke, Robert J. Francis, Evgenia Tsaousi, Colin Longstaff

**Affiliations:** 1 Biotherapeutics, National Institute for Biological Standards and Control, S Mimms, Herts, UK; 2 Biological Imaging Group, Analytical Biological Sciences, National Institute for Biological Standards and Control, S Mimms, Herts, UK; 3 School of Biological Sciences, University of Essex, Colchester, UK

**Keywords:** Cell death, Histocytochemistry, Biochemistry, Cell biology

## Abstract

Neutrophils are pivotal players in immune defence which includes a process of release of histones and DNA as neutrophil extracellular traps (NETs). Histones, while toxic to invading pathogens, also kill host cells, including neutrophils. Bacteria have evolved mechanisms to escape neutrophils, including the secretion of leucocidins (e.g. ionomycin). Live cell video microscopy showed how fibrinogen and fibrin influence NETosis and neutrophil responses to extracellular histones. Histones were rapidly lethal to neutrophils after binding to cells, but formation of fibrinogen/fibrin-histone aggregates prevented cell death. Histone cytotoxicity was also reduced by citrullination by peptidyl arginine deiminase 4, or digestion by serine proteases. Ionomycin and phorbol 12-myristate 13 acetate (PMA) are used to trigger NETosis. Fibrinogen was responsible for a second distinct mechanism of neutrophil protection after treatment with ionomycin. Fibrinogen clustered on the surface of ionomycin-stimulated neutrophils to delay NETosis; and blocking the β integrin receptor, α_M_β_2,_ abolished fibrinogen protection. Fibrinogen did not bind to or protect neutrophils stimulated with PMA. Fibrinogen is an acute phase protein that will protect exposed cells from damaging circulating histones or leucocidins; but fibrinogen depletion/consumption, as in trauma or sepsis will reduce protection. It is necessary to consider the role of fibrinogen in NETosis.

## Introduction

Since the discovery of neutrophil extracellular traps (NETs) as a host defence mechanism capable of trapping and killing bacteria^1^, there has been a growing interest and research output^2^. It is generally accepted that the process of generating NETs, NETosis, is a specific type of programmed cell death involving changes in the nucleus and nuclear membrane, followed by cell membrane breakdown and expulsion of DNA and associated proteins, including histones, neutrophil elastase and myeloperoxidase^3^. NETs appear to generate a physical barrier and range of biochemical weapons against many pathogens, including bacteria, fungi, viruses and parasites, and are triggered by diverse pathways in the neutrophil^4^. Reactive oxygen species (ROS) are implicated in NET formation and a distinction is drawn between NADPH-oxidase (NOX2) dependent and -independent mechanisms (though it is important to remember that mitochondria can also produce reactive oxygen species (ROS) in NOX2-independent NETosis^4^). Common triggers used to induce neutrophils to generate NETs in vitro are phorbol 12-myristate 13 acetate (PMA) and leucocidin calcium ionophores such as ionomycin. These chemicals trigger distinct pathways^5–7^ leading to the release of different families of proteins with different patterns of post translational modification^8^. Whilst PMA is considered to induce typical NOX2-dependent NETosis and ionomycin-induced NETs are NOX2-independent, the relationship between NETs produced in vitro by these triggers and NETosis in vivo is unclear. Furthermore, there are many areas of disagreement and conflicting findings in the field, arising from different methodologies including NET triggers, cell source (e.g. human versus murine) and protocols^2,4,7,9^.

It is generally agreed that while NETs appear to be an important form of innate immune defence, they are a double-edged sword and can potentially result in damage to the host^10^. There is a link between NETs and the generation of common and debilitating autoimmune diseases such as rheumatoid arthritis and lupus, amongst others^4,8^ and circulating nucleosomes, DNA or histones are associated with disease^11–13^. In some cases problems arise from the ability of DNA networks to enhance clot formation and stability, which may be countered by treatment with DNases^12,14–16^. However, histones, especially when free from DNA, have long been recognised as highly cytotoxic and are seen as important biomarkers to chart progress of diseases including sepsis, acute lung diseases and thrombotic disorders, and are targets for therapy^17–19^. The role of histone citrullination during the formation of NETs is an area of controversy. The process is seen by some as an essential early step of chromatin decondensation and integral to classical NETosis^20–23^, but as not essential for NETosis by others^7,24–26^. Histone citrullination, by peptidylarginine deiminase 4 (PAD4), is a deimination reaction which modifies arginine residues and reduces protein net positive charge. This presents a conundrum since charge density is seen as an important component in the bactericidal activity of histones and citrullination inevitably reduces their potency^27,28^. So, there is a question around why neutrophils deactivate the weapons apparently released during NETosis. This picture is further complicated by recent findings that neutrophils express PAD4 on their surface and release another citrullinating enzyme, PAD2, into the local environment^29^. The ability of PAD4 to work at all in the low intracellular Ca^2+^ environment has been questioned, leading to a conclusion that typical NETosis, as triggered by PMA for example, does not require PAD4 activity^24^.

Histones interact with many biomolecules, including nucleic acids, proteins and glycosaminoglycans. Charge is important as histones are rich in arginine and lysine residues, but they also contain hydrophobic domains. The structure of the nucleosome that is released during NETosis contains histones bound to DNA by charge-charge interactions, but the histone core, consisting of H2A, H2B, H3 and H4 is also bound together by hydrophobic interactions^30^. H1 and H5 are ‘linker’ histones and not as tightly bound to the nucleosome, so may be more likely to circulate freely. Histones are known to bind to fibrinogen and a study by Gonias et al.^31^ investigated in detail the ‘paracoagulant’ behaviour of H3 towards fibrinogen which results in gel formation. However, the impact of fibrinogen and fibrin on histone activity around NETs is poorly understood.

Furthermore, fibrinogen and fibrin are intimately linked with the behaviour of leukocytes, for example via integrin receptors α_M_β_2_ (CD11b/CD18, Mac-1, CR3) and α_X_β_2_, which are significant regulators of inflammatory responses and neutrophil survival^32,33^. NETs in vivo are closely associated with fibrin clots and neutrophils are bathed in fibrinogen, which is present in circulation at around 3 mg/ml. The focus of the current work is to understand how fibrinogen and fibrin affect neutrophil survival during NETosis, and how these proteins interact with histones, which are at the same time a potent weapon in the neutrophil armoury against invading pathogens and an agent of host cell damage.

## Results

### Histone cytotoxicity

**Figure 1:**
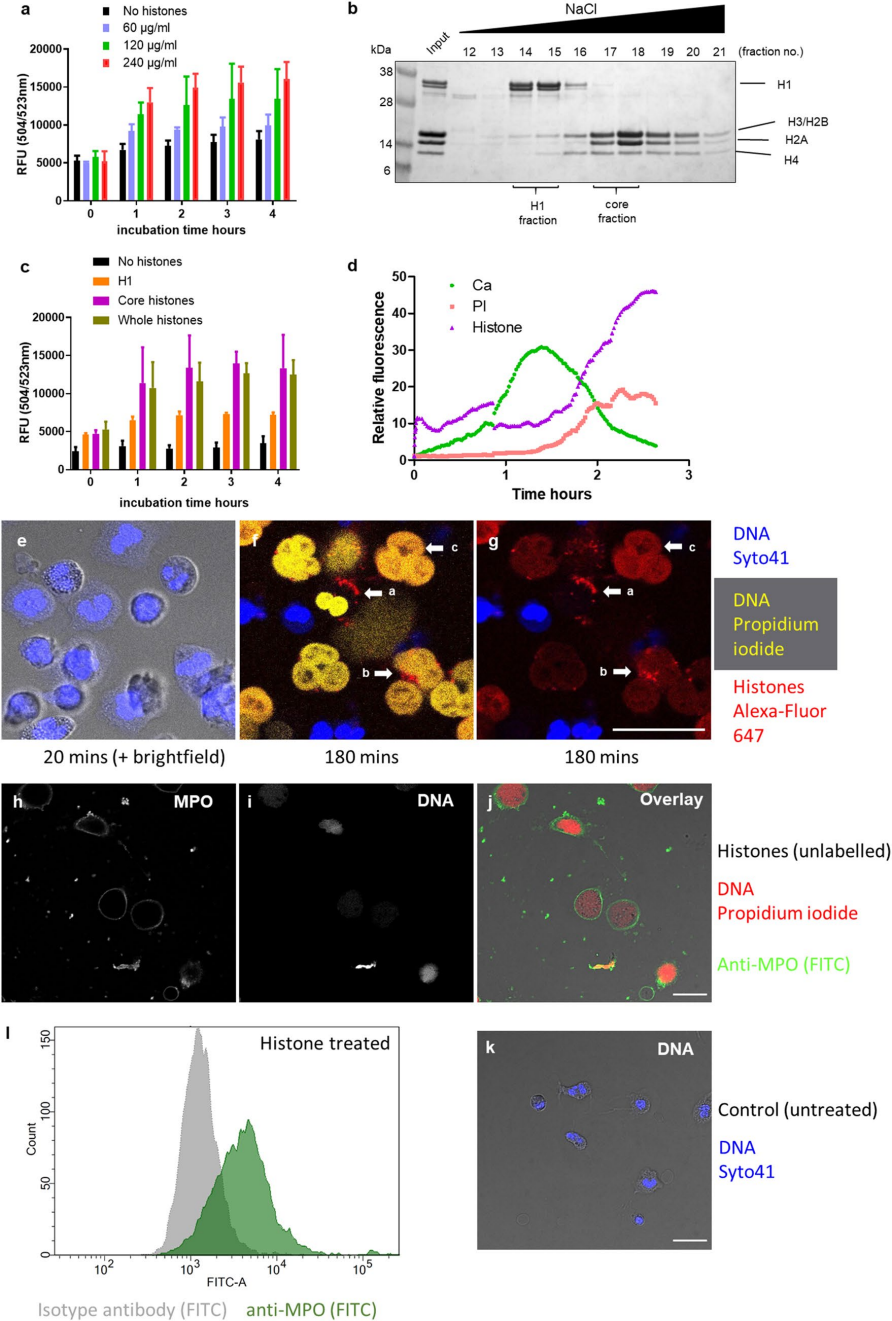
Cytotoxicity of extracellular histones. (**a**) Time course of Sytox Green neutrophil viability assays with increasing concentrations of mixed histones. Neutrophils were seeded in 96 well plates and allowed to adhere before media was removed and replaced with media containing the indicated concentration of mixed histones and Sytox Green. Fluorescence was measured every hour in a plate reader. The fluorescent signal is proportional to cell death, error bars show 95% confidence intervals (CI) for the mean, n = 3. (**b**) Mixed histones were fractionated using heparin-Sepharose chromatography to separate core histones (H2, H3 and H4) from H1 and analysed by SDS-PAGE and Coomassie staining. (**c**) Time course for mixed and fractionated histones (60 μg/ml) incubated with neutrophils in a Sytox Green cell viability assay. (**d**) Traces from live cell microscopy experiments that included Fluo-4 to follow intracellular Ca^2+^, propidium iodide for extracellular DNA and histones labelled with Alexa-Fluor 647. Neutrophils were plated onto a glass-bottomed dish in media containing Syto41 and propidium iodide before addition of labelled histones. An early rise in intracellular Ca^2+^ was followed by increased fluorescence signal for propidium iodide and histones as they bound to exposed DNA. (**e**–**g**) Images from a live cell microscopy time lapse video of mixed labelled histones incubated with neutrophils. Dyes used were Syto41 for intracellular DNA (blue), 20 µg/ml Alexa-Fluor 647 labelled histones (red) and propidium iodide for exposed DNA or DNA in cells with compromised membranes (yellow). (**e**) 20 min incubation showing the lobular structure of neutrophil nuclei. (**f**) 3 h incubation showing cell surface histone binding and many dead cells (propidium iodide positive). (**g**) The same image as (**f**) without the propidium iodide channel to highlight histone staining in red. Cell ‘a’ had histones bound to the cell surface and damage has progressed to the stage where propidium iodide accessed and stained intracellular DNA (yellow in (**f)**). Labelled histones appear within cell ‘b’, indicating cell membrane damage sufficient to allow histones to enter. Structure ‘c’ is expelled DNA with bound histones, following cell membrane disintegration. The scale bar is 25 µm. Representative images from 1 of n = 3 independent experiments. (**h**–**j**). Neutrophils were incubated with histones for 3 h before addition of FITC-labelled anti-myeloperoxidase (MPO) antibody and propidium iodide to visualise DNA. (**h**) and (**i**) are individual channels for MPO and DNA, respectively. (**j**) is the merged image of (**h**) and (**i**). (**k**) Untreated neutrophils stained with Syto41 (DNA) for comparison. Images show expanded/decondensed DNA in the histone treated cells with externalised MPO. Scale = 20 μm. Representative images of n > 50 cells in at least 5 fields of view/sample. (**l**) Flow cytometric quantitation of MPO externalisation in histone treated (120 μg/ml) neutrophils shows high levels of MPO release similar to that previously observed with ionomycin treatment^62^. Anti-MPO FITC (green) is compared with isotype control (grey).

Figure 1 summarises the toxicity of mixed and fractionated histones towards neutrophils. Sytox Green assays show increasing fluorescence as intracellular DNA becomes accessible due to loss of membrane integrity. Figure 1a illustrates a dose response, and after fractionation (Fig. 1b), it is apparent that lysine and arginine rich core histones (H2A, H2B, H3 and H4) are more toxic than H1 (Fig. 1c). The mechanism underlying histone toxicity is revealed in Fig. 1d–g and Supplementary Figure S1. Fluo-4 provides a signal for intracellular Ca^2+^ which increased after binding of Alexa-647-labelled histones, followed by cell death, and increasing propidium iodide fluorescence (Fig. 1d and S1). The later decline in Fluo-4 signal in Fig. 1d may be explained by breakdown of the cell membrane to release Ca^2+^ and Fluo-4 into the medium (while increasing exposure of DNA resulted in further propidium iodide and histone binding). Live cell microscopy of neutrophils treated with 20 µg/ml mixed Alexa-647-labelled histones (red) further elaborated the mode of action. Images from time lapse video recording of neutrophils incubated with labelled histones are shown in Fig. 1e–g and initially show healthy multi-lobular nuclei which then gradually lost definition to appear grey as histones bound to the cell. After 3 h (Fig. 1f–g) histones were seen as red puncta on the membrane, coinciding with entry of propidium iodide staining the nucleus yellow (cell ‘a’). Ultimately, large NET-like DNA structures that are stained with both propidium iodide and labelled histones were apparent (marked ‘b’ and ‘c’). Confirmation that these structures were histone-induced NETs is provided in Fig. 1h–l, showing positive staining for myeloperoxidase (MPO) by imaging and flow cytometry.

### Fibrin(ogen) protects neutrophils from histone cytotoxic effects

**Figure 2:**
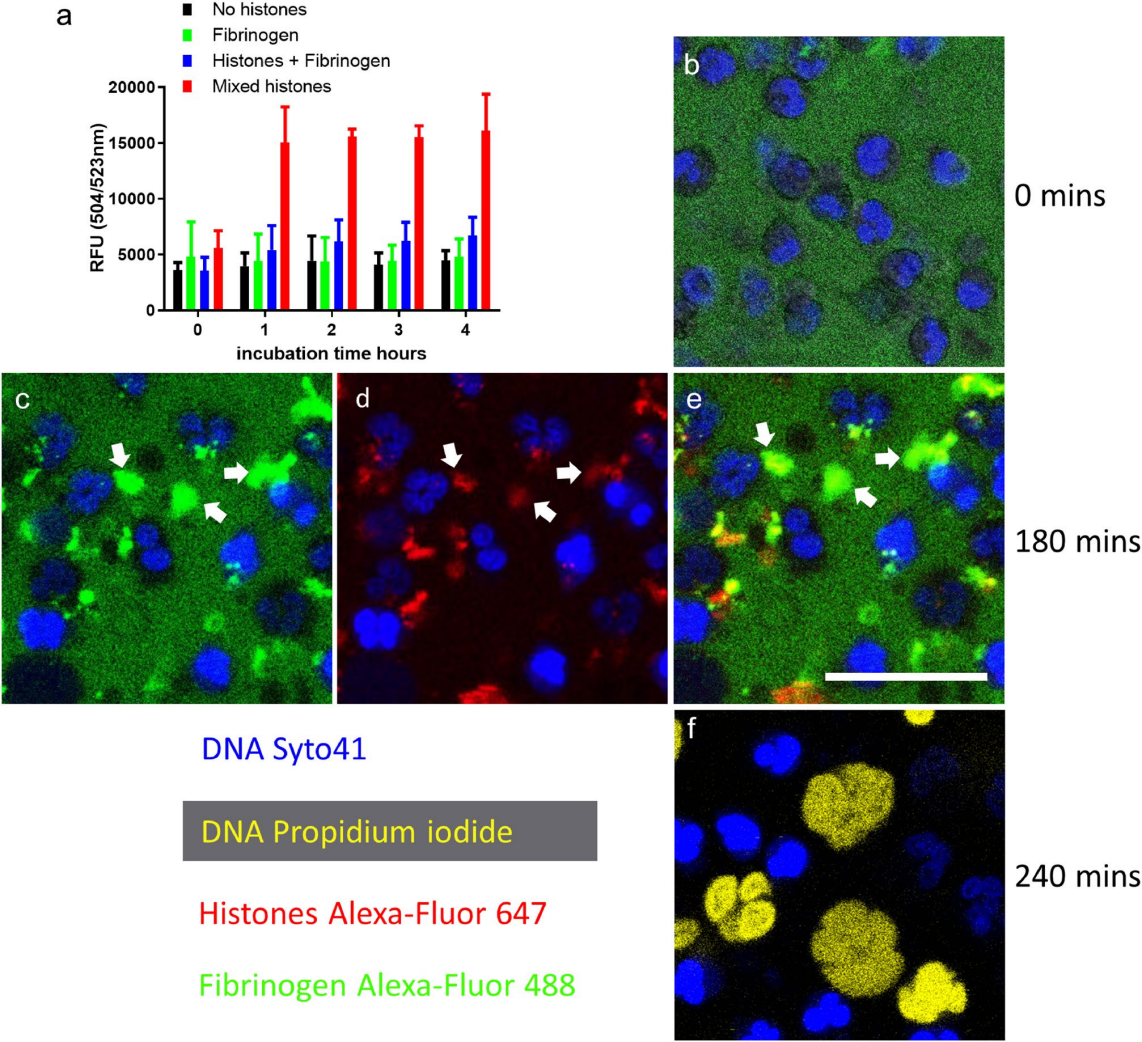
Fibrinogen protects neutrophils from histone cytotoxicity. (**a**) Sytox Green neutrophil viability assays. Neutrophils were seeded in 96 well plates and allowed to adhere before media was removed and replaced with media containing histones and/or fibrinogen and Sytox Green. Fluorescence was measured every hour. Cells were stable in media and with added fibrinogen, error bars show 95% CI for the mean, n = 3. Cells were efficiently killed with 60 µg/ml of mixed histones within an hour but were rescued by the presence of 0.5 mg/ml fibrinogen. Panels (**b**–**e**) are stills from live cell videos of neutrophils to explore the mechanism of fibrinogen protection. Neutrophils were plated onto a glass-bottom dish in media containing Syto41 and propidium iodide before sequential addition of fibrinogen and histones. (**b**) Neutrophils against a green background of labelled 2.5 mg/ml fibrinogen (spiked with Alexa 488-fibrinogen) with no histones added at time zero. (**c**–**e**) Cells after 180 min of incubation with Alexa-Fluor 647 labelled histones (20 μg/ml) in media containing Alexa-Fluor 488 labelled fibrinogen and showing Syto41 blue stained nuclei. Images shown are (**c**) green/blue channels, (**d**) red/blue channels and (**e**) merged image. Rapid aggregate formation between histones and fibrinogen was apparent, which persisted (e.g. arrows). There were many intact cells, retaining the lobular nuclear structure of healthy neutrophils, stained in blue with Syto41, even after 180 or 240 min of incubation under these conditions. (**f**) An image taken after 240 min showing some large externalised NET-like DNA structures stained yellow with propidium iodide. The scale bar is 25 µm. Representative images are shown from 1 of n = 2 independent experiments.

Fibrinogen protected neutrophils from the cytotoxic effects of histones, as demonstrated in Fig. 2. Histones were again rapidly lethal (within 1 h) towards neutrophils (Fig. 2a) but neutrophils maintained good viability at 4 h in the presence of fibrinogen. Live cell microscopy studies with 20 µg/ml mixed Alexa-Fluor 647-labelled histones (red), and Alexa-Fluor 488-labelled fibrinogen (green), revealed a likely mechanism. Fibrinogen, initially seen as a diffuse background (Fig. 2b) rapidly formed stable aggregates with histones (Fig. 2c–e, arrows). By 4 h approximately one third of cells had died under these conditions, to produce large NET-like DNA structures (Fig. 2f), like those seen in Fig. 1f,g.

**Figure 3:**
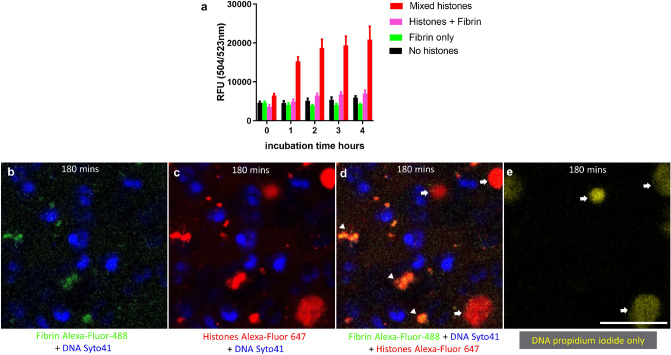
Protection of neutrophils from histone cytotoxicity by fibrin. (**a**) Sytox Green neutrophil viability assays were performed as in Fig. 2, with fibrin (as a suspension of FDP) in place of fibrinogen. Fibrin protected cells from the cytotoxic effects of histones. Error bars show 95% CI for the mean, n = 3. (**b-e**) Live cell microscopy studies were performed by adding fibrin (derived from Alexa-Fluor 488-labelled fibrinogen in green) and Alexa-Fluor 647 labelled histones (red) to neutrophils in a glass-bottomed dish. Images were taken after 180 min of incubation. (**b**) fibrin and (**c**) histone fluorescence with (**d**) as the merged image to highlight complexes between fibrin and histones (arrow heads). (**e**) Is the propidium iodide signal only. White arrows in (**d**) and (**e**) highlight some binding of extracellular histones to DNA released from damaged cells. The scale bar is 25 µm. Representative images are shown from 1 of n = 3 independent experiments.

Fibrin was formed by treatment of purified fibrinogen (spiked with Alexa-Fluor 488-fibrinogen for live cell imaging work) in the presence of FXIIIa and plasminogen, and subsequently digested by addition of uPA and tPA to make a heterogeneous suspension of fibrin-degradation products (FDP). Figure 3a, shows that fibrin, like fibrinogen, was also protective against histones and Fig. 3b–d suggest the same underlying mechanism of complex formation accounts for protection by fibrinogen or fibrin, which is histone complex formation. Figure 3d is the merged image from the green and red channels and arrowheads highlight colocalization of histones and fibrin. The arrows in Fig. 3d,e highlight the colocalization of propidium iodide and histones with large external DNA, NET-like structures.

### Fibrinogen-histone complex formation

**Figure 4:**
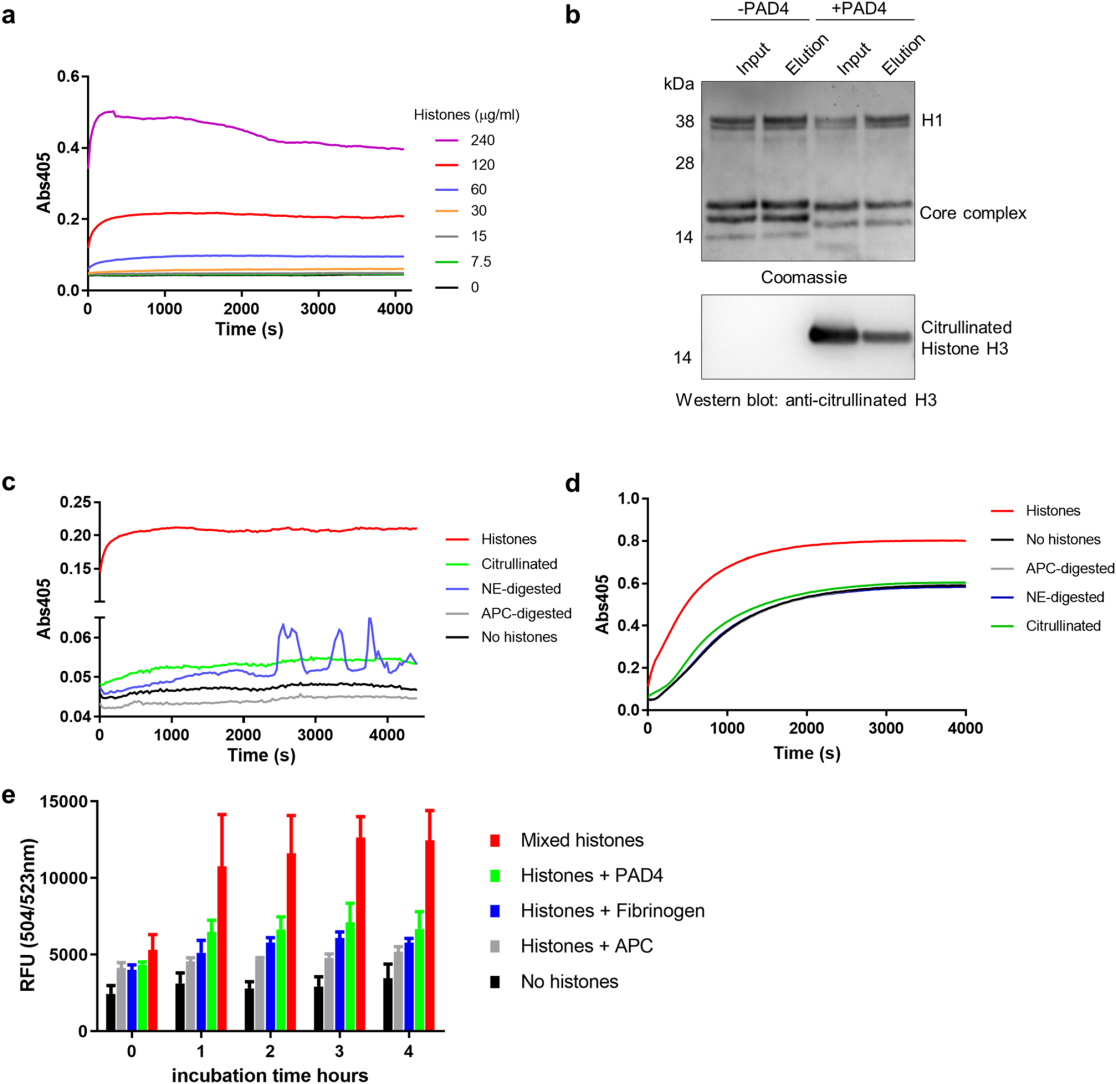
Effects of histones and modified histones on fibrinogen gelation, clotting and cytotoxicity. (**a**) Concentration dependent fibrinogen gel formation in the presence of mixed, unmodified histones from 0 to 240 µg/ml with 2.8 mg/ml fibrinogen. (**b**). Mixed histones were treated with PAD4 and purified by heparin-Sepharose chromatography and the western blot confirms efficient citrullination of histone H3. (**c**) Prevention of gelation by histone modification with PAD4, or following digestion with proteases, neutrophil elastase (NE) or activated Protein C (APC). (**d**) Similar results to (**c**) were observed where clotting of fibrinogen-histone mixtures was triggered by thrombin. (**e**) Histone modification reduced the cytotoxicity of histones towards neutrophils, as shown by results from Sytox Green cell viability assays. Neutrophils were plated in 96 well plates and allowed to adhere before media was removed and replaced with media containing the indicated concentration of modified or unmodified histones, fibrinogen, and Sytox Green. Fluorescence was measured every hour. Error bars show 95% CI for the mean, n = 3.

Mixtures of histone and fibrinogen have been observed to form precipitates or gels and the process can be studied optically, as shown in Fig. 4a. The effects of histone modification were also explored in Fig. 4. The enzyme PAD4 is associated with NET formation and chromatin de-condensation by catalysing arginine deimination with the loss of positive charge. Figure 4b confirms by western blotting of citrullinated H3 that PAD4 was able to efficiently modify our histone preparation, and Fig. 4c shows that the treatment with PAD4, or histone digestion with neutrophil elastase, or activated Protein C (APC) compromised the interaction between histones and fibrinogen. This loss of interaction was replicated in experiments to investigate fibrinogen clotting by thrombin, Fig. 4d, which showed a lack of stimulation of fibrin formation by modified histones. Figure 4e, summarises the decrease of histone cytotoxicity effected by citrullination or proteolysis. Therefore, while the ability of fibrinogen to defend host cells against histones was compromised by histone proteolysis or posttranslational modifications that occur as a result of NETosis, the modified histones produced were also less cytotoxic. Furthermore, as citrullinated histones did not promote fibrinogen gelation or accelerate thrombin activity, they should be less prothrombotic in vivo.

### Fibrin(ogen) effects on neutrophils treated with ionomycin or PMA

**Figure 5:**
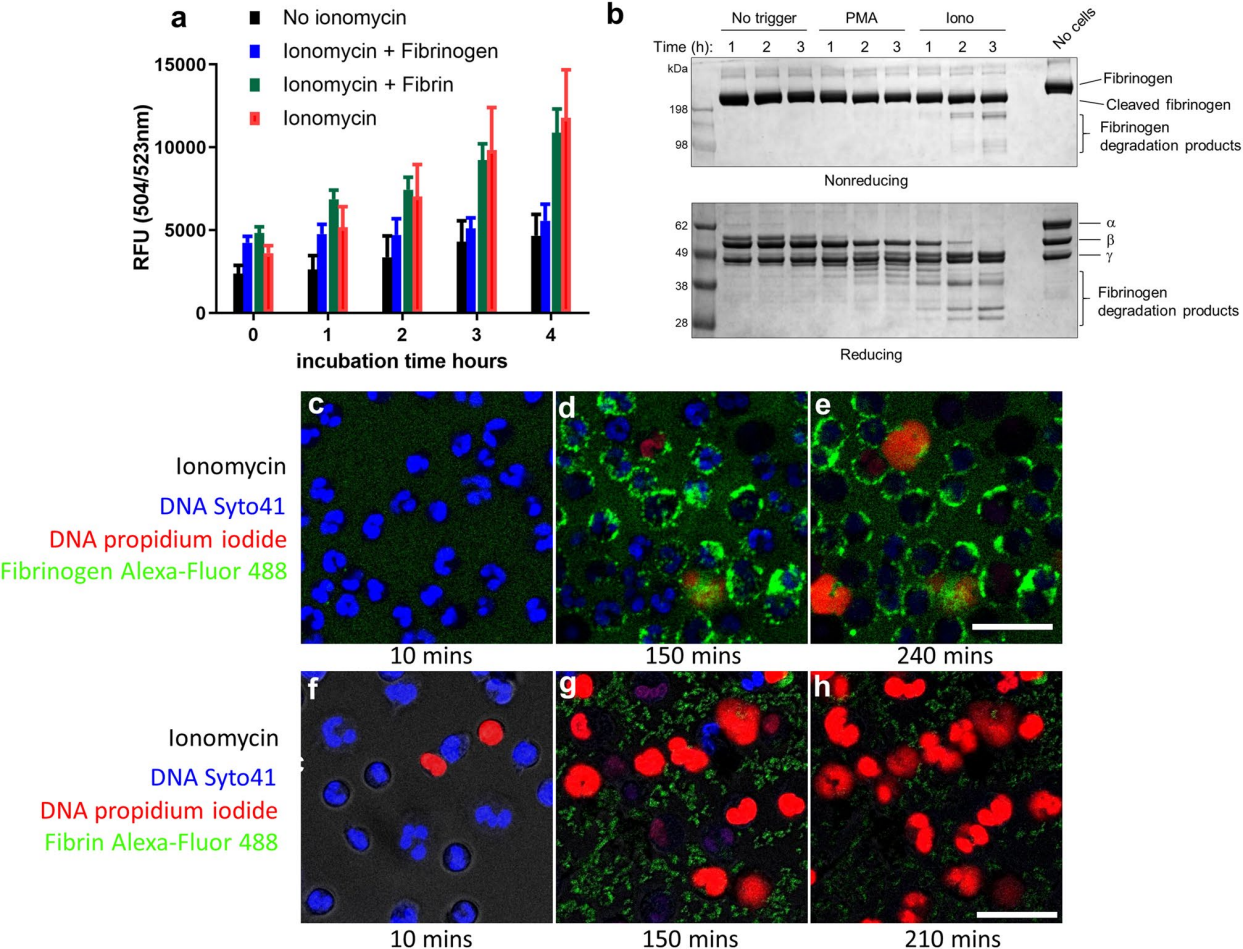
Fibrinogen delays NETosis by neutrophils treated with ionomycin, fibrin does not. (**a**) Sytox Green cell viability assays indicate that cell survival after treatment with ionomycin was improved in the presence of fibrinogen but not by fibrin. Neutrophils were seeded in 96 well plates and allowed to adhere before media was removed and replaced with media containing ionomycin with or without fibrinogen/fibrin. Fluorescence was measured every hour. Error bars show 95% CI for the mean, n = 3. (**b**) SDS-PAGE (non-reducing and reducing) and Coomassie staining analysis of fibrinogen (present at 2 mg/ml) from neutrophil supernatants after 1, 2, or 3 h with no treatment, or treatment with PMA or ionomycin (Iono). There was a rapid loss (within 1 h) of the fibrinogen α chain under all conditions, but ionomycin treatment resulted in most fibrinogen proteolysis. The fibrinogen γ chain was most resistant. (**c**–**e**) Images taken at 10, 150 and 240 min from live cell microscopy time lapse videos of neutrophils treated with ionomycin in the presence of Alexa-Fluor 488 fibrinogen (green) and propidium iodide to stain externalised DNA (red). By 150 min (**d**) there was widespread cell-binding and clustering of fibrinogen, which persisted up to 240 min (**e**), and was accompanied by improved survival as seen by the low level of propidium iodide staining or NET formation. (**f**–**h**) Results from a parallel experiment with ionomycin treated neutrophils in the presence of fluorescent fibrin rather than fibrinogen. Early and progressive staining of DNA with propidium iodide is apparent and was widespread by 150 min, by which time many of the cells were dead. The scale bar is 25 µm. Representative images are shown from 1 of 3 independent experiments for both fibrinogen and fibrin.

Experimentally, NETs are often induced using PMA or calcium ionophores such as ionomycin and typical NET formation is shown in Supplementary Fig. S2. These results highlight some of the morphological differences between NETs formed by these triggers under our conditions. Ex vivo studies on NETosis with neutrophils are often performed in culture medium or in the presence of serum, so the influence of fibrinogen and fibrin will be overlooked but warrants investigation. The results shown in Fig. 5 again suggest a crucial role for fibrinogen in prolonging neutrophil survival after ionomycin treatment. Interestingly, fibrin was not effective in this system (Fig. 5a). Rapid proteolysis of fibrinogen was seen in the presence of neutrophils, untreated or treated with PMA and was even more marked after ionomycin treatment (Fig. 5b). The α chain was rapidly lost in all situations; and the γ chain most resistant. The low level of NETosis with ionomycin + fibrinogen demonstrated in Fig. 5a was replicated in the time lapse videos, as seen in images Fig. 5c–e. In contrast, the initiation of NETosis was not delayed by fibrin, as shown by the images in Fig. 5f–h with many cells staining with propidium iodide after 150 and 210 min, indicating progression to NETosis (also in line with Fig. 5a). An early event, taking place at 1–2 h after ionomycin treatment, was binding and clustering of labelled fibrinogen to the neutrophil cell surface, and this was maintained over the 5 h of the experiment. No corresponding cell surface binding of fibrin was seen.

**Figure 6:**
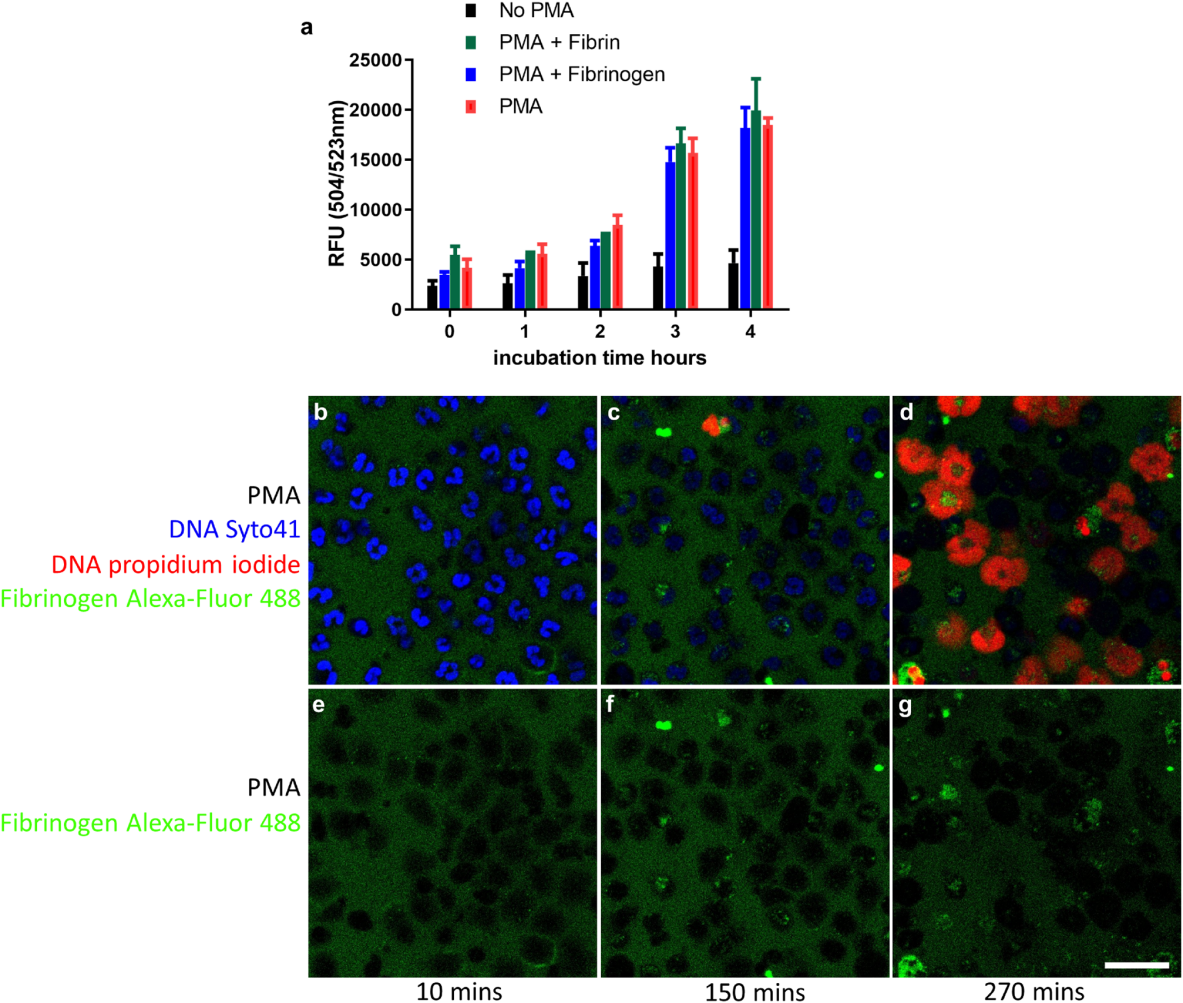
Neither fibrinogen nor fibrin delay NETosis in neutrophils treated with PMA. (**a**) Sytox Green cell viability assays over time after treatment with PMA (100 nM), showed lack of protection by fibrinogen or fibrin. Assays were performed as in Fig. 5, with PMA in place of ionomycin. Error bars show 95% CI for the mean, n = 3. (**b**–**d**) There was no change in the course of NETosis triggered by PMA effected by fibrinogen as shown by images from live cell microscopy videos. Release of DNA NETs can be seen by propidium iodide signal in red. (**e**–**g**) The same images as (**b**–**d**) showing only the green fluorescent channel to focus on the lack of cell surface fibrinogen binding (compare with fibrinogen binding after ionomycin treatment in Fig. 5). The scale bar is 25 µm. Representative images are shown from 1 of 3 independent experiments.

The parallel experiment treating cells with PMA in place of ionomycin was also performed and results are summarised in Fig. 6. In this case the Sytox Green cell viability assay demonstrated no effect of fibrin or fibrinogen on cell death (Fig. 6a). Figure 6b–d shows images from live cell time-lapse videos of PMA-treated neutrophils in the presence of labelled fibrinogen (c.f. Fig. 5c–e). No cell surface-fibrinogen binding is apparent and by 270 min abundant NETs can be seen. The lack of binding and clustering of fibrinogen is highlighted in Fig. 6e–g, which is the green channel only of Fig. 6b–d. Comparison of Fig. 6d,g suggests some clustering of fibrinogen with exposed DNA/histones or other cell debris after cell death.

### Phosphatidylserine (PS) exposure

**Figure 7:**
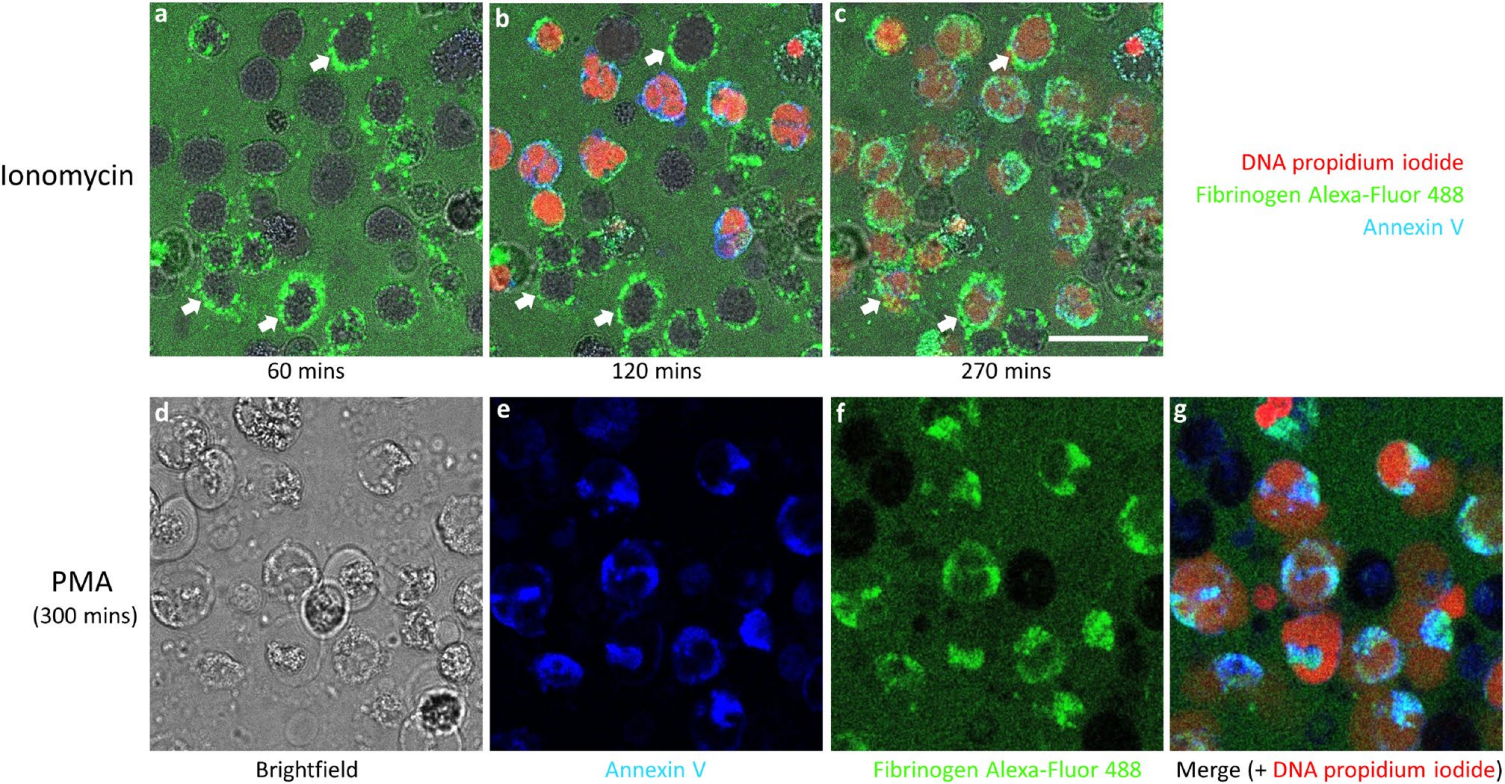
Fibrinogen interactions with cells treated with ionomycin or PMA in the presence of annexin V. Live cell confocal microscopy was performed on neutrophils treated with ionomycin in the presence of Alexa Fluor 488-labelled fibrinogen and propidium iodide (red for accessible or extracellular DNA) and Pacific Blue-labelled annexin V. (**a**–**c**) Images captured after 60, 120 and 270 min. (**a**) Good surface binding and clustering of fibrinogen by approximately half the cells at 60 min. (**b**) By 120 min the cells without bound fibrinogen were stained with Pacific Blue-labelled annexin V and at the same time internal DNA was accessible to propidium iodide indicating the cell membranes were losing integrity. (**c**) After 270 min most cells were stained with propidium iodide. (**d**–**g**) Images from neutrophils 300 min after treatment with PMA, including (**d**) a brightfield image, (**e**) annexin V binding, (**f**) fibrinogen binding, and (**g**) a merged image also including propidium iodide staining of DNA in red. After PMA treatment there was no fibrinogen interaction with the cells before NETosis occurred, but fibrinogen bound to the residual membrane fragments after NETosis and was coincident with annexin V binding. The scale bar is 25 µm. Representative images are shown from 3 independent experiments.

To investigate the relationship between PS exposure and fibrinogen-neutrophil binding (Fig. 5), experiments were performed in the presence of Alexa-Fluor 488 labelled fibrinogen and annexin V-Pacific Blue and are shown in Fig. 7. It is apparent from Fig. 7a–c that once again fibrinogen bound rapidly after ionomycin treatment, but before there was any obvious annexin binding (Fig. 7a). There was a heterogeneous response of the cells for fibrinogen binding, so that some cells bound fibrinogen (e.g. those marked by arrows), while others did not (Fig. 7a–c). At 120 min the cells without bound fibrinogen stained with annexin V and had lost membrane integrity as shown by propidium iodide staining, indicating the cells had become leaky (Fig. 7b). By 270 min (Fig. 7c) most cells stained heavily for fibrinogen, propidium iodide and annexin V, though had not disintegrated. Once again, no fibrinogen binding was observed after PMA treatment of neutrophils and no annexin V binding was seen before NETosis (not shown). Ultimately, binding of fibrinogen and annexin V to cell membrane debris did take place after NETosis had occurred, as shown in Fig. 7d–g. Annexin binding in these experiments is associated with loss of membrane integrity and is delayed on cells treated with ionomycin that bind fibrinogen.

### Integrin receptor α_M_β_2_ (CD11b/CD18, Mac-1, CR3)

**Figure 8:**
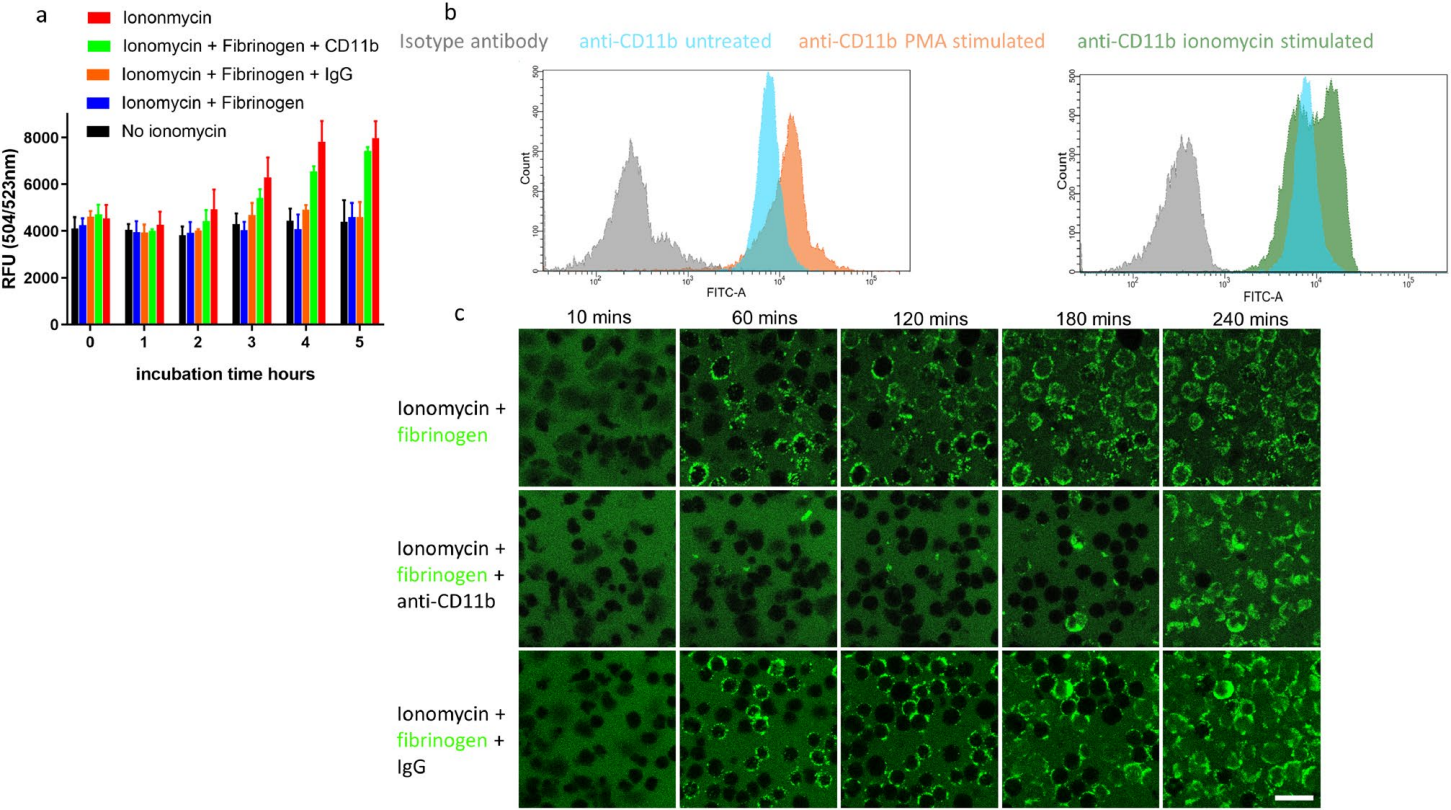
Blocking the integrin receptor α_M_β_2_ prevents fibrinogen binding and the delay of NETosis triggered by ionomycin. (**a**) Results from Sytox Green cell viability assays over 5 h where NET formation, DNA release and cell death was triggered by ionomycin. Neutrophils in 96 well plates were incubated with ionomycin supplemented with the indicated combinations of fibrinogen and anti-CD11b antibody (or isotype control), together with Sytox Green. Fluorescence was measured every hour. Cell survival is promoted by the presence of fibrinogen, but this effect is blocked by the addition of anti-CD11b (but not isotype control antibody, IgG). Error bars show 95% CI for the mean, n = 3. (**b**) Flow cytometry was used to investigate the presence of CD11b (part of the α_M_β_2_ integrin receptor). Untreated neutrophils reacted with FITC-anti-CD11b indicating the presence of the receptor (blue histograms) and treatment with PMA (orange) or ionomycin (green) increased the receptor signal. (**c**) Images from live cell confocal microscopy where neutrophils were treated with ionomycin in the presence of Alexa-Fluor 488-labelled fibrinogen (green). Substantial amounts of surface binding and clustering of fibrinogen were seen developing from 60 min (top row), which appeared to be blocked in the presence of 10 µg/ml of anti-CD11b (middle row). The final image at 240 min with anti-CD11b shows fibrinogen interacting with membrane fragments from the many dead cells at this timepoint (as in Fig. 7f). The bottom row of images includes an isotype control IgG antibody and gives similar results to those seen in the top row. Representative images are shown from 1 of 2 independent experiments that included antibodies or 3 independent experiments without antibodies.

As fibrinogen is a known ligand for neutrophil receptor α_M_β_2_, it was of interest to see what effect blocking the receptor would have on fibrinogen binding to ionomycin treated neutrophils. Representative results are shown in Fig. 8, including an anti-CD11b monoclonal antibody. As expected, fibrinogen again prolonged survival after treatment with ionomycin, but protection was reversed by pre-treatment of cells with the antibody to CD11b (Fig. 8a). These results strongly suggest that fibrinogen acts via the α_M_β_2_ receptor to delay NETosis induced by ionomycin. It is noteworthy that the receptor was present in unstimulated cells and increased after ionomycin or PMA treatment, as shown by flow cytometry results (Fig. 8b). Here, binding of FITC-labelled anti-CD11b to unstimulated cells (blue histograms), indicates the presence of surface α_M_β_2_, and anti-CD11b binding increased with either PMA (orange histograms) or ionomycin treatment (green histograms), where there seemed to be two populations of α_M_β_2_-positive neutrophils. Live cell microscopy supported the notion that α_M_β_2_-fibrinogen binding after ionomycin treatment could be blocked by anti-CD11b (Fig. 8c). The final time point in Fig. 8c shows some fibrinogen aggregation, but this is most likely binding to cell membrane fragments arising from dead cells, as seen in Fig. 7f.

## Discussion

Fibrinogen and fibrin have been overlooked in research on NETs in in vitro and ex vivo experiments, which are often conducted in cell culture medium with or without added serum, so lacking fibrinogen. Here we report on two distinct mechanisms where fibrinogen or fibrin protect neutrophils during NETosis. These are (1) protection by fibrinogen or fibrin of cells from the cytotoxic effects of released histones by sequestering them as histone-fibrin(ogen) complexes; (2) the delay by fibrinogen (but not fibrin) of NETosis triggered by ionomycin (but not PMA). These observations are of interest as variations in fibrinogen levels occur during disease and may influence progression and outcomes. For instance, fibrinogen is an acute phase protein and the circulating concentration increases during infections and inflammatory conditions so that it may become more effective at neutralising histones or interacting with neutrophils. Conversely, in sepsis and disseminated intravascular coagulation, or following trauma, for example, fibrinogen levels often fall, and free histones may become more potent as a result. It is also known that bacteria express or release many fibrinogen binding proteins^34^ to coat themselves with host fibrinogen, for example streptococcal M1 protein^35^. It is speculated that this acts as a defensive cloak against host immune recognition, but microbially-bound fibrinogen could also act as a shield to bind and detoxify host-generated histones.

Histones released from damaged cells, whether following necrosis or NETosis, constitute Danger (or Damage) Associated Molecular Patters (DAMPs or alarmins) that trigger a variety of host responses. Some of these may be harmful, including an excessive inflammatory response leading to damage to many cell types (endothelium, epithelium, kidney, lung, heart, pancreas, brain)^18,19^. The cytotoxicity of histones raises the possibility of local or disseminated collateral damage or a ‘self-sustaining cascade’ or ‘positive feedback loop’ of damage^36^, which would amplify the original DAMP signal. The in vitro results presented here show that neutrophils are susceptible to damage by histones, but crucially the vicious cycle of histone release and cytotoxicity may be ameliorated by fibrinogen or fibrin and their degradation products. In vivo, the distinct roles of free histones, histones bound to DNA and nucleosomes and their cytotoxic and immunostimulatory mechanisms are incompletely understood. The release of nucleosomes and different histone subtypes, the action of serum nucleases on nucleosomes, the modification of histones by proteases and peptidylarginine deiminase (PAD) enzymes are dynamic processes that present challenges to our understanding. Circulating DNases may play a significant role in destroying NETs^14^, thus complicating their identification and quantification in vivo, whilst also providing a mechanism for the release of free histones.

The findings presented here using labelled histones show rapid binding at the neutrophil cell surface leading to increased permeability and Ca^2+^ entry followed by reorganisation of the cell nucleus and release of DNA structures that resemble NETs. This behaviour has been observed previously with isolated neutrophils where histones were able to induce release of NET-like structures (as induced by PMA, for example), and cause myeloperoxidase release (a NET marker) but not generation of ROS^37^. Further evidence that histones are NET triggers was also provided here by detection of myeloperoxidase release in flow cytometry work and the images shown in Fig. 1h–l. The mechanism of histone-cell binding that leads to NETosis may be through direct phospholipid interactions or via receptors. For example, histones are known to interact with cells via Toll Like Receptors (TLR, including TLR2, -4 and -9)^38–40^, and there is evidence that binding via this mechanism stimulates NET generation. Huang et al^41^ studying ischemia/reperfusion injury in the liver, in mice, observed dose dependent increases in markers of NETosis in response to histones which involved neutrophil TLR4 and TLR9. These authors proposed that histones released from stressed hepatocytes could stimulate neutrophils to form NETs to exacerbate liver damage. TLR involvement was also proposed as a mechanism in a study on acute kidney injury where histones released from necrotic cells could induce NET formation to accelerate kidney damage, promote inflammation and trigger remote organ injury in the lungs by further NET formation^42^. In both these cases anti-histone treatments were effective in reducing injury severity.

An alternative mechanism of histone-cell interaction proposes direct histone binding to the cell membrane, possibly via exposed phosphatidylserine (PS) or phosphatidylethanolamine (PE)^37^, to form cationic pores allowing Ca^2+^ influx and cell death. Other studies have demonstrated that histone binding to cultured endothelial cells could be blocked by liposomes containing PC, PS and PE^43^; or demonstrated direct binding of H1, H5 and H4 to lipid bilayers^44^. Recently, a detailed study showed how histone H4 induces pores in cell membranes in a charge dependent manner that allowed propidium iodide influx^45^, as seen in the current work. We observed that more highly charged core histones were more damaging than H1, and the range of histone concentrations used in our studies, usually 20 µg/ml in live cell microscopy, were within those observed in other studies. For example, in trauma in humans or mouse models^37^, or from recent mass spectrometry analysis of plasma from sepsis patients where H3 was measured up to 60 µg/ml^46^, and within the range seen in animal models of sepsis^47^. Although healthy cells would have little PS exposed on the outer membrane leaflet, it has been suggested a low level of initial histone binding may trigger further binding by enhancing expose of PS and/or PE to accelerate membrane damage and increased Ca^2+^ influx^48^. It is possible that histone binding to TLRs and directly to cell membranes are both involved in NET formation, but this question requires further investigation. Whatever the mechanism involved, our results show fibrinogen or fibrin provided protection against histones by delaying cell death and NET release.

Loss of charge density by citrullination or proteolysis, reduced histone toxicity towards neutrophils, further suggesting charge involvement and in agreement with previous results on other cell types^28,49^. Charge density has long been implicated in the antibacterial effects of histones^27^ and it is known that polycationic peptides are toxic to bacteria and eukaryotic cells. The cytotoxicity of histones has made them a therapeutic target using polyanions such as non-anticoagulant heparinoids^50,51^, or anti-histone antibodies^37^ or APC to proteolyse histones (which may not be effective in all situations^49^). C reactive protein (CRP) has also been proposed as a natural defence against circulating histones and has been found to bind and detoxify core histones and H1 in a variety of systems involving cultured endothelial cells, platelets and animal models^43^.

There is ample evidence of histone interactions with fibrinogen and fibrin from turbidity studies, biophysical studies (isothermal titration calorimetry and small angle X-ray scattering) and proteomics approaches^31,52^. Results presented in Fig. 4 support these earlier findings and show that histones accelerate clotting in the presence of thrombin. Neutralisation of histone toxicity by fibrinogen was clear from Sytox Green assays, and fibrin(ogen)-histone complex formation is the likely mechanism according to results from live cell microscopy outlined in Figs. 2 and 3. Recent modelling work on fibrinogen has identified an uneven distribution of negative and positive charged areas on the D- and E-domains, which could provide binding sites for histones. It may be the case that interactions between histones and fibrinogen are more than simply electrostatic^31^, unlike histones and polysialic acid or heparin and other glycosaminoglycans, for example. It is not known how histone-fibrinogen complexes are related to histone-CRP complexes^43^, but it should be noted that fibrinogen is always present in the circulation at much higher concentrations than CRP, normally around 3 mg/ml compared with < 3 µg/ml for CRP. Based on these many lines of evidence it is reasonable to propose that fibrinogen can act as a buffer against the cytotoxic effects of histones in vivo. The neutralising effects of fibrinogen and fibrin against histones may be under-appreciated in in vitro experiments in cell culture medium, with or without added serum.

Detailed observations of neutrophils stimulated with common triggers such as PMA, calcium ionophores, bacteria or fungi have shown different pathways to NETosis^7^ and variations in the morphology of the NETs produced^5,6^, which were also noted in Fig. S2 comparing NETs produced by PMA or ionomycin. Recent proteomics studies have confirmed that PMA and ionomycin trigger release of distinct groups of NET-associated proteins^8^. For example, PMA stimulation produced more H3 while A23187 (a calcium ionophore) led to a greater release of PAD4 and H1 and more citrullination^8^, suggesting a less cytotoxic histone environment in this case. Although PMA and ionomycin are widely used as in vitro triggers of NETosis, there is some controversy around what biological processes they are mimicking. Konig and Andrade^24^ have argued that PMA and calcium ionophores induce completely different pathways. According to this view, PMA would reproduce defensive NETosis against pathogens, whereas ionomycin initiates a process used by bacteria for self-protection. In this way, calcium ionophores or bacterial pore forming toxins are leucocidins that trigger ‘leukotoxic hypercitrullination’, which is defined as Ca^2+^ stimulation of PAD4 activity that does not involve NOX2 activation or ROS generation^24^. The strong activation of PAD4 and subsequent modification of histones would be consistent with a mechanism to reduce their bactericidal potency. Another possible role of citrullination as a neutrophil defensive mechanism is possible following recent findings that PAD4 is present on the neutrophil outer cell surface and the related enzyme, PAD2 is secreted into the local environment by neutrophils^29^. These authors have suggested that pericellular citrullination is a ‘brake’ to detoxify histones and prevent collateral damage that could lead to widespread host cell death, where neutrophils would be in the front line.

Differences in fibrin(ogen) binding behaviour of neutrophils after PMA or ionomycin treatment were highlighted in the current work as shown in Figs. 5, 7 and 8. Ionomycin treatment led to a rapid (1–2 h) association of labelled fibrinogen with the cell surface in a proportion of cells, which was accompanied by improved cell survival. Heterogeneity of neutrophil populations is well known, and ageing, which is accompanied by increased CD11b expression, may be involved^53^. The results shown in Fig. 7 show a distinction between cells that bind annexin and those that bind fibrinogen after ionomycin treatment. Previous studies have investigated fibrinogen interactions with CD11b/CD18 on neutrophils and subsequent signalling pathways that lead to delayed apoptosis^33^. Notably in the current work, only ionomycin treatment led to clear fibrinogen binding and delayed NETosis.

Fibrinogen is a known ligand of the neutrophil integrin receptor α_M_β_2_ (Mac-1, CD11b/CD18, CR3) but is generally seen as a poor ligand when intact and in solution^54^. Previous microscopy studies showing ‘macro-clustering’ of α_M_β_2_ in the presence of pro-survival, anti-apoptotic ligands, including fibrinogen, have produced images similar to those presented here in Figs. 5, 7 and 8^55^. Binding and clustering of fibrinogen was only seen after ionomycin treatment in the current work. The lack of binding of fibrin suspensions to neutrophils in our experiments is interesting given the promiscuous behaviour of α_M_β_2_ towards many ligands, including known binding sites on fibrinogen α, β and γ chains^56^. Prasad and colleagues have attempted to study the relative importance of fibrinogen versus fibrin in host defence mechanisms against *S. aureus* intraperitoneal infection^57^. By using a fibrinogen variant that could not clot, they concluded that fibrin formation was important for clearing infection and survival, but fibrin formation alone was insufficient, and fibrin-integrin binding was more important than fibrinogen binding. In our experimental systems we cannot rule out a complete lack of binding of fibrin to ionomycin treated neutrophils. The bulk concentration of fibrin binding sites would be lower in the fibrin suspension used, due to their internal masking within fibrin fibres compared to soluble fibrinogen molecules at the same protein concentration. Hence it may be more difficult to observe fibrin binding. However, fibrin was not effective in the Sytox Green assay system (Fig. 5a), supporting a dominant role for fibrinogen.

The results shown in Fig. 5b suggest that fibrinogen in the presence of neutrophils in our experimental systems would be partially degraded, and especially so after ionomycin treatment. An important binding site of the α_M_β_2_ receptor on the fibrinogen D-domain is located on the γ-chain residues 390–396^58,59^, also the chain most resistant to neutrophil-associated proteolysis (Fig. 5b). Conformational changes in fibrinogen are able to enhance receptor interactions, for example after adsorption on surfaces, but proteolytic degradation has a role in unmasking binding sites in the D-domain to facilitate interaction with α_M_β_2_^54,60^. For example, recent studies have proposed that full length fibrinogen interacts only weakly with α_M_β_2_, whereas fungal protease-derived fibrinogen cleavage products (termed ‘cryptokines’), form a complex with α_M_β_2_ and TLR4 and play a role in antifungal immune defence^60^. Whatever fibrinogen structures are binding to α_M_β_2_ after ionomycin treatment, the details of the signalling events are not known. Presumably there is ‘inside-out’ signalling in neutrophils leading to conformational changes and activation of α_M_β_2_, and possibly externalisation of additional internal receptor stores and interactions with other receptors. According to our results, the α_M_β_2_ present on unstimulated neutrophils or after stimulation with PMA does not bind fibrinogen (Fig. 8).

More work is needed to understand all the implications of the current work in situations where NETosis is taking place in vivo. Details of the activation of α_M_β_2_, fibrinogen binding and subsequent signalling, and the structural changes in fibrinogen, fibrinogen degradation products and fibrin that regulate binding to integrin receptors, require further investigation. Nevertheless, these studies show that fibrinogen is involved in modulating the behaviour of neutrophils towards bacterial leucocidins and can protect cells from host-derived histones. We suggest the role of fibrin(ogen) warrants greater attention in studies on NETosis.

## Materials and methods

### Materials and reagents

IIA mixed calf thymus histones, PMA (Phorbol 12-myristate 13-acetate), and recombinant PAD4 were from Sigma (Poole, Dorset, UK). Sytox Green, Ionomycin, Annexin V Pacific Blue, Alexa Fluor 488 labelled fibrinogen, Syto41, Fluo-4 intracellular Ca^2+^ reporter and Alexa Fluor 647 labelling kit were from Molecular Probes (ThermoFisher Scientific, Waltham, MA, USA). Fibrinogen was from Calbiochem (Nottingham, UK). Anti-CD11b (clone M1/70, ab8878) and isotype control (ab18536) were from Abcam (Cambridge, UK). FITC-labelled anti-CD11b (clone M1/70) and isotype control were from ThermoFisher Scientific (Waltham, MA, USA). Anti-MPO FITC (clone MPO421-8B2) and isotype control were from Biolegend (San Diego, CA, USA).

### Histone separation and modification

IIA mixed histones were diluted in HEPES-buffered saline (HBS, 10 mM HEPES pH 7.4, containing 150 mM NaCl) and applied to a HiTrap heparin-Sepharose column using an AKTA purifier (both GE Healthcare, Hatfield, UK). Histones were eluted over a continuous salt gradient (0–2 M NaCl) and fractions analysed by SDS-PAGE and Coomassie staining. This provided a means to separate the core complex from the H1 histone. Appropriate fractions were pooled and dialysed into HBS. Protein concentrations were determined with the Pierce BCA protein assay kit (ThermoFisher). Histone citrullination was performed by incubating histones (600 μg/ml) with recombinant PAD4 (25 nM) in citrullination buffer (10 mM HEPES pH 7.4, 4 mM CaCl_2_, 4 mM DTT) for 16 h at 37 °C. Citrullinated histones were purified using HiTrap heparin-Sepharose chromatography as above, except that elution was performed in a single step with 2 M NaCl. Citrullination was confirmed by western blotting with anti-citrullinated histone H3 antibodies (ab5103, Abcam, Cambridge, UK). For digestion reactions, histones (600 μg/ml) were incubated with 250 nM activated protein C (APC, Xigris, Eli Lilly Indiana IN, USA), or 10 nM neutrophil elastase (Sigma Poole, Dorset, UK) for 16 h at 37 °C, and purified using HiTrap heparin-Sepharose, as described above.

### Fibrinogen gelation and fibrin polymerisation

Fibrinogen (2.8 mg/ml) was mixed with histones (0–240 μg/ml) in microtitre plates (100 μl reaction volume), and the turbidity monitored at 405 nm every 30 s at 37 °C in a Spectramax M5 plate reader (Molecular Devices, Stanford, CA, USA). To determine the effects of histones on fibrin formation, 1 nM thrombin (code 01/578, NIBSC, S. Mimms, UK) was added to the reaction mix.

### Neutrophil isolation

Blood was obtained with informed, signed consent from healthy local donors following approval by the NIBSC ethics committee (Human Materials Advisory Committee). All procedures used in the current work conformed to local rules and were in accordance with UK Human Tissue Act regulations. Neutrophils were isolated from heparin anti-coagulated blood by dextran sedimentation followed by Ficoll-Hypaque density centrifugation (GE Healthcare) according to Nauseef^61^, and maintained in RPMI 1640 media (minus phenol red, Gibco, ThermoFisher).

### Sytox Green assays

Neutrophil viability was quantified in a high-throughput manner with the cell-impermeable DNA-binding dye Sytox Green. Modes of cell death were distinguished by the timing and magnitude of the Sytox Green signal. Histone treatment resulted in rapid membrane damage and Sytox Green fluorescence (< 1 h), whereas PMA/ionomycin treatment resulted in later peak fluorescence (3–4 h) which coincided with the detection of NET DNA from cells as the membrane ruptured. These timings were complemented and confirmed by direct visualisation of morphological changes to cellular DNA using live cell imaging, described below and elaborated in Supplementary Fig. S2.

Isolated neutrophils were seeded in Corstar clear bottom, black walled 96 well plates (Corning Inc Kennebuck, ME, USA) at a density of 2.5 × 10^5^ cell/well in RPMI 1640 media (minus phenol red). Cells were allowed to adhere for 45 min at 37 °C in a humidified incubator with 5% CO_2_. Media was removed and the cells treated with combinations of histones (fractionated, digested, citrullinated, or intact, 0–240 μg/ml), fibrinogen (0.5 mg/ml), FDPs (0.5 mg/ml), PMA (100 nM), Ionomycin (5 μM), in RPMI 1640 media also containing 5 μM Sytox Green. Blocking antibodies to CD11b (clone M1/70) or IgG isotype control, were used at 10 μg/ml. Cells were incubated at 37 °C in 5% CO_2_ and the fluorescence monitored every hour in a Spectramax M5 plate reader (Molecular Devices) using emission/excitation wavelengths of 504/523 nm. Results are presented as bar graphs showing different conditions and time points and include 95% confidence intervals to estimate statistically significant responses between bars.

### Live cell imaging

Approximately 5 × 10^6^ neutrophils were seeded onto a glass-bottomed dish (World Precision Instruments, Hitchin, UK). Upon commencement of live cell imaging 20 µg/ml histones (Alexa Fluor 647 labelled or unlabelled), PMA (100 nM), Ionomycin (5 µM) or fibrinogen or fibrin (2.5 mg/ml spiked with 75 µg/ml Alexa-Fluor 488 fibrinogen, unless otherwise stated) were added in sequential order, with fibrinogen/FDPs first and then PMA/Ionomycin/histone stimulus.

Viable cells were visualised with the cell permeable DNA-binding dye Syto41 (10 µM, Thermo Scientific) and dead/damaged cells with the cell impermeable DNA-binding dye propidium iodide (0.5 µg/ml, Biolegend, San Diego, CA, USA). Annexin V Pacific Blue (Thermo Scientific) was used at 5 µl per 1 × 10^6^ cells.

To visualise myeloperoxidase (MPO) on histone-stimulated neutrophils, neutrophils were seeded (2 × 10^5^) onto glass-bottomed dishes (Ibidi) and incubated with histones (120 μg/ml) for 3 h before addition of FITC-labelled anti-MPO antibody and propidium iodide or Syto41, as described.

To investigate the effects of CD11b blocking, CD11b antibody (clone M1/70), or isotype control, were pre-incubated with the neutrophils in suspension 15 min before seeding onto the glass bottomed dish, at 10 μg/ml.

Time-lapse videos were acquired on a TCS SP8 X confocal laser-scanning microscope (CLSM) (Leica Microsystems, Wetzlar, Germany) at a rate of 1 frame every 2 min.

### Flow cytometry

Flow cytometry conditions were as report previously^62^. Briefly for histone treatment, isolated neutrophils (2 × 10^6^) were seeded onto polystyrene 4-well dish (Thermofisher) and incubated with histone (120 μg/ml) in RPMI media for 3 h. Neutrophils were resuspended in 1 ml fresh media. A 79 µl aliquot was stained with FITC conjugated anti-MPO antibody (clone MPO421-8B2) or isotype control (20 µl) (BioLegend cat. No. 347201), and propidium iodide (1 µl of 1 mg/ml) (Sigma). A further 400 µl of media was added prior to analysis. Samples were run on a BD FACS Canto II (BD, Wokingham, UK) with appropriate compensation controls. Data was analysed using BD FACS Diva software. For PMA and ionomycin stimulation isolated neutrophils (2 × 10^6^) were seeded onto a dish and incubated with stimulus for 30 min. The neutrophils were then resuspended and stained with anti-CD11b FITC antibody (clone M1/70) and propidium iodide (0.5 µg/ml, Biolegend, San Diego, USA). Samples were run on a BD FACS Canto II (Wokingham, UK). Live neutrophils were identified through gating of forward and side scatter, and then negative for propidium iodide. At least 10,000 events of live neutrophils were recorded per condition.

### Fibrin (as fibrinogen degradation products, FDPs)

FDP suspensions were used to represent fibrin and digested fibrin that may be present in vivo. Heterogeneous but reproducible mixtures of FDPs of different sizes were generated from purified fibrinogen (Calbiochem) clotted with thrombin (NIBSC code 01/578) and pre-activated factor XIII (FXIII, NIBSC, code 02/170), lysed in the presence of tissue plasminogen activator (tPA, NIBSC, code 98/714) or urokinase plasminogen activator (uPA, NIBSC, code 11/184) and glu-plasminogen or lys-plasminogen (Hyphen Biomed, and Immuno, Vienna, Austria, respectively). Where present, Alexa Fluor 488 was at 1:55 ratio with fibrinogen. The reaction mixture for clot lysis was made by mixing 0.2 ml of mixture A, containing thrombin, FXIIIa, CaCl_2_, tPA or uPA, with 1 ml of mixture B containing, fibrinogen and plasminogen. Final concentrations were: 4 nM thrombin, 2.5 mg/ml fibrinogen, 5 mM CaCl_2_, 1 IU/ml FXIIIa, 2 nM tPA or 9 nM uPA and 220 nM plasminogen. Samples of the lysing fibrin mixture were taken at 4 time points for each set of reaction conditions at the time the clot collapsed, t_lysis_ (around 10 min), t_lysis_ + 5 min, 60 min and 300 min. Reaction samples were mixed with excess aprotinin and hirudin to inhibit plasmin and thrombin, respectively, and placed on ice before mixing and snap freezing.

## Supplementary information

Supplementary information.
