# Supplementary material for: Fibrinogen protects neutrophils from the cytotoxic effects of histones and delays neutrophil extracellular trap formation induced by ionomycin
Source: Sci Rep. 2020 Jul 16;10:11694. doi: 10.1038/s41598-020-68584-0 (PMC7366688; doi:10.1038/s41598-020-68584-0)
Supplement: Supplementary file 1 — Supplementary information. [file 41598_2020_68584_MOESM1_ESM.pdf]

Supplementary Figures

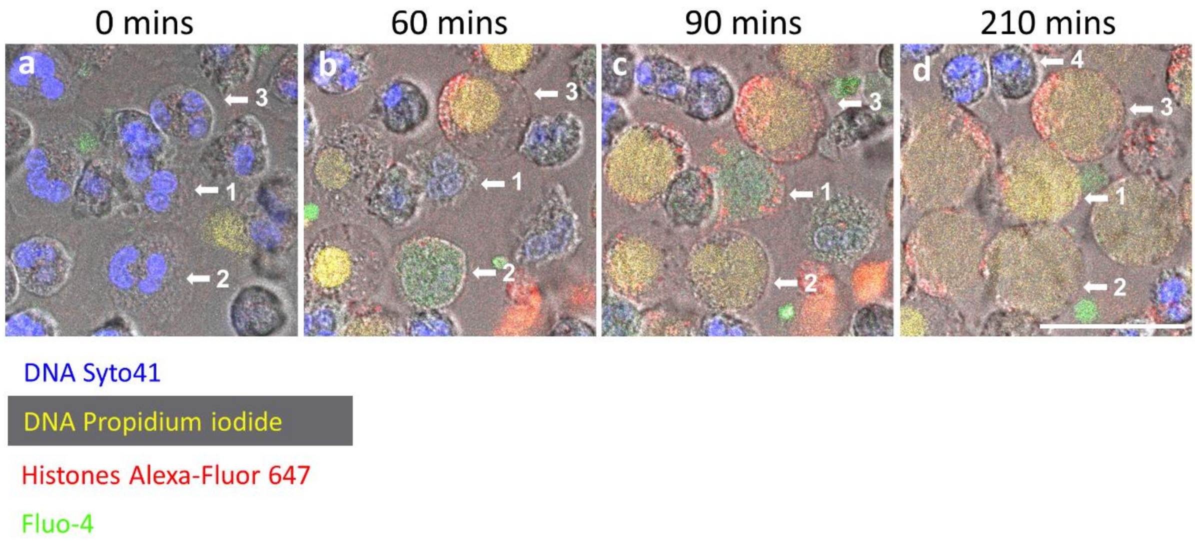

Figure S1. Cytotoxicity of extracellular histones. Live cell microscopy time lapse video showing histone binding to neutrophils and subsequent effects on intracellular Ca^{2+} and cell death. Neutrophils were seeded onto a glass-bottomed dish in media containing cell permeable Syto41 (blue, to detect DNA in intact healthy cells), cell impermeable propidium iodide (yellow, to detect extracellular DNA or DNA in cells with permeable or damaged membranes) and Fluo-4 (green, to detect intracellular Ca^{2+} increase). Alexa Fluor 647 labelled histones (red, 20 μg/ml) were added to the cells and monitored over time. Panel (a), at the outset of the experiment, neutrophils are characterised by their bilobed nucleus (blue). Panel (b), after 60 mins, cell (1) has yet to bind histones, cell (2) has histones on the cell membrane (red speckled) which coincides with elevated intracellular Ca^{2+} (green). Cell (3) has histones bound to the membrane and become permeable to propidium iodide (yellow nucleus), indicating membrane damage. Panel (c), after 90 mins, the nuclear material of cells (2) and (3) have decondensed and expanded to fill the cytoplasm. Cell (1) now has histones bound to the cell membrane, characterised by red puncta, and elevated intracellular Ca^{2+} (green). Panel (d), after 210 minutes, nuclei of cells with bound histones have completely broken down and DNA is beginning to be released (cell (1)). Cells are greatly expanded compared to 0 mins or compared to neighbouring cells without bound histones (e.g. cell (4) in panel (d)). Scale bar is 25 μm.

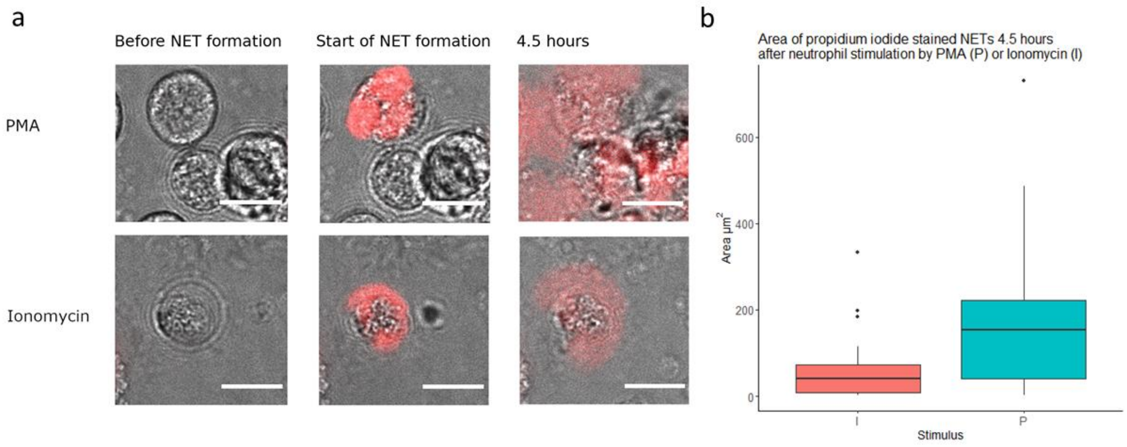

c

Stimulus	Median	IQR	Count	P value
Ionomycin	40.8	7.6 – 80.9	25	0.02
PMA	153	34.3 - 227	25	

Figure S2. Treatment of neutrophils with PMA or ionomycin. (**a**) Bright field images overlaid with propidium iodide staining (red) follow the development of NETs after neutrophils were treated with PMA or ionomycin. The earliest images show some loss of nuclear structure before DNA staining with propidium iodide. NETosis progresses with extrusion of DNA and propidium iodide staining (some staining was routinely seen within 30 min, without added fibrinogen). After 4.5 hours those cells destined for NETosis had completed the process. The NET area grew over this period and staining became more diffuse. (**b**) The areas of propidium iodide positive NETs were calculated from a random selection of cells (n=25) after 4.5 hours and the distributions are summarised as a boxplot. (**c**) A summary of the analysis of the areas including median and interquartile range (IQR). The NET areas were not normally distributed (Shapiro-Wilk tests $p<0.001$ both PMA and Ionomycin distributions) and non-parametric testing using the Mann-Whitney-Wilcox test gave a low probability that the area distributions were equivalent ($p = 0.02$). It can be concluded that PMA treatment generally resulted in a larger NET structure than ionomycin treatment.
